# *Metschnikowia pulcherrima* and Related Pulcherrimin-Producing Yeasts: Fuzzy Species Boundaries and Complex Antimicrobial Antagonism

**DOI:** 10.3390/microorganisms8071029

**Published:** 2020-07-12

**Authors:** Matthias Sipiczki

**Affiliations:** Department of Genetics and Applied Microbiology, University of Debrecen, 4032 Debrecen, Hungary; gecela@post.sk

**Keywords:** *Metschnikowia*, taxonomy, barcodes, genome, reticulation, antagonism, pulcherrimin, iron-depletion, bioprotection

## Abstract

Yeasts affiliated with the *Metschnikowia pulcherrima* clade (subclade) of the large ascomycetous genus *Metschnikowia* frequently turn out to produce the characteristic maroon-red pulcherrimin when tested for pigment production and prove to exert antagonistic effects on many types of microorganisms. The determination of the exact taxonomic position of the strains is hampered by the shortage of distinctive morphological and physiological properties of the species of the clade and the lack of rDNA barcode gaps. The rDNA repeats of the type strains of the species are not homogenized and are assumed to evolve by a birth-and-death mechanism combined with reticulation. The taxonomic division is further hampered by the incomplete biological (reproductive) isolation of the species: certain type strains can be hybridized and genome sequencing revealed chimeric genome structures in certain strains that might have evolved from interspecies hybrids (alloploid genome duplication). Various mechanisms have been proposed for the antimicrobial antagonism. One is related to pulcherrimin production. The diffusible precursor of pulcherrimin, the pulcherriminic acid is secreted by the cells into the environment where it forms the insoluble pulcherrimin with the ferric ions. The lack of free iron caused by the immobilization of ferric ions inhibits the growth of many microorganisms. Recent results of research into the complexity of the taxonomic division of the pulcherrimin-producing *Metschnikowia* yeasts and the mechanism(s) underlying their antimicrobial antagonism are discussed in this review.

## 1. Introduction

**Figure 1:**
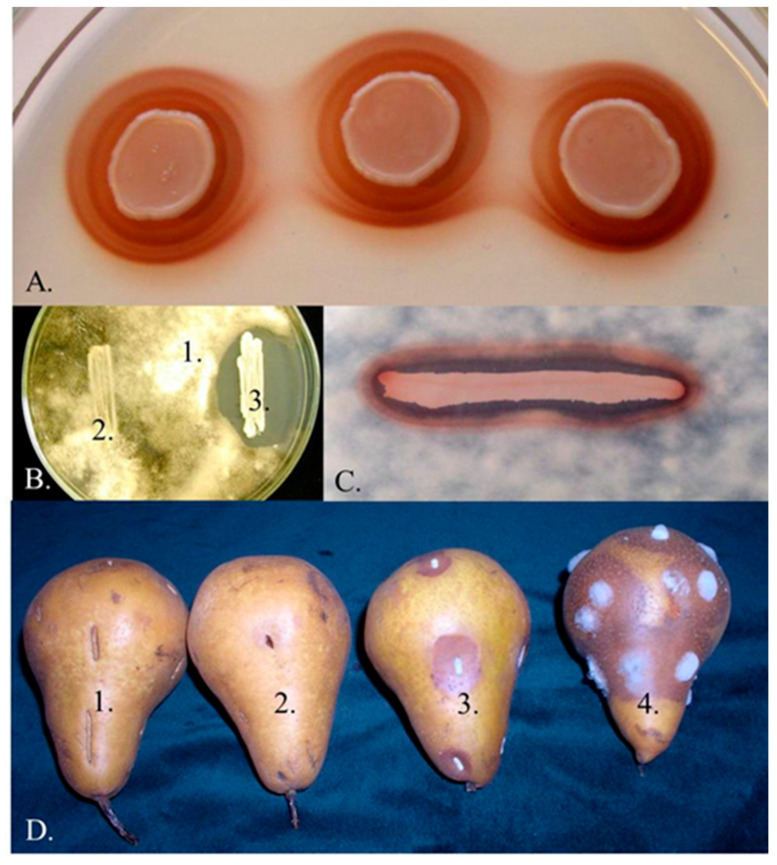
Pigment production and antagonism. (**A**) Pigmented halos around the colonies of the *Metschnikowia* strain 02.11.1.21. (**B**) Inhibition zone around the colony of the same strain (1: *Botrytis cinerea* 3318, 2: *Saccharomyces cerevisiae* S288c, 3: *Metschnikowia* 02.11.1.21). (**C**) Coincidence of pigmented halo and inhibition zone around the colony of *Metschnikowia* 02.11.1.21 on a medium supplemented with 0.005 mg/mL FeCl_3_ and flooded with conidia of *B. cinerea* 3318. (**D**) Inhibition of rotting caused by *Botrytis* (1: untreated, 2: dipped in a suspension *Metschnikowia* 02.11.1.21 cells, 3: dipped in a mixed suspension of *B. cinerea* 3318 conidia and *Metschnikowia* 02.11.1.21 cells, 4: dipped in a suspension of *B. cinerea* 3318 conidia). See reference [6] for the description of the strains.

*Metschnikowia* Kamienski (1899) is a large ascomycetous genus currently comprising 79 species (Mycobank, 04. 2020) but the number or species is continuously growing. The *M. pulcherrima* clade of the genus contains seven validly described species that share the ability to produce pulcherrimin, a maroon-red pigment (reviewed in [1,2]). These species and the strains closely related to them have broad biotechnological potential for application in various industrial processes. In wine fermentation, these yeasts can modulate the population dynamics of the fermenting yeast communities and produce enzymes and a broad range of compounds that improve the aromatic complexity of the wine (for a review, see [3]). Due to the large fatty globules in their chlamydospores (“pulcherrima cells”), the strains are outstanding candidates for low-cost lipid production (reviewed in [4]). Their most intensively studied property is the strong antimicrobial activity (Figure 1A–C) (e.g., [5,6,7,8,9,10,11,12]). Since the mechanisms underlying their antagonistic effect are not associated with the production of toxic compounds, these strains can safely be used as bioprotective agents to curb the invasion of pathogenic and rotting (saprophytic) microorganisms (Figure 1D) and/or additives in food technologies to modulate the dynamics of microbial populations. Numerous technological innovations involving antagonistic *Metschnikowia* strains have been patented (e.g., JPH01117778A, 1989; US6991930B1, 2006; NZ528225A, 2008; P0800775, 2008; ITTO20070655A1, 2009; WO2010149370, 2010; WO2010149369, 2010; CN101946805A 2011; CN103642705A, 2014; EP3266305A1, 2018; CN107904180A, 2018; CN110684678A, 2020;) and several *Metschnikowia-*based products have been commercialiZed [Excellence Bio-Nature (Lamothe-Abiet), Flavia and Gaïa (Lallemand), Shemer (Bayer, Koppert Biological Systems) Zymaflore Egide (Laffort)] as ADYs (Active Dry Yeast) for inoculated fermentation or as biocontrol agents for application against plant pathogens and post-harvest plant diseases. 

Over the past two decades, large numbers of strains isolated from various substrates have been assigned to one or the other of these species (preferentially to *M. pulcherrima*) on the basis of barcode sequence (preferentially the D1/D2 domains of the LSU rRNA genes and the ITS1-5.8S-ITS2 segments of the rDNA repeats) identities/similarities. The general practice of sequence-based strain identification is a search with the sequence of the strain in nucleotide databases for identical/similar sequences and assigning the strain to the species whose database sequence is found most similar. The sequence of the strain is then routinely deposited in the database (most journals request accession numbers) under this taxonomic name usually without an expert taxonomic verification. Since small sequence differences are usually tolerated during identification, the new entries will gradually fill up the barcode gaps separating the closely related species; the species boundaries gradually become fuzzy. Thus, the taxonomic identification of new isolates by comparing their rDNA sequences with those deposited in databases can easily lead to false results. In addition to this general problem, other difficulties can also arise when pulcherrimin-producing *Metschnikowia* strains are to be identified taxonomically. The results of the in-depth analyses of the rDNA repeats of certain type strains [13,14], the hybridization of type strains with each-other [14] and the analysis of genome sequences (e.g., [15,16,17]) raised doubts as to whether the taxonomic division of the *M. pulcherrima* clade is correct at all. The incongruences around the mechanism of the antimicrobial antagonism pose another problem. While many researchers associate it with iron depletion, others prefer the view that non-iron-related mechanisms similar to those known in other antagonistic microorganisms are also involved or even play the major role. This review seeks to provide an overview of the recent results of research into the complexity of the taxonomic relationships among the pulcherrimin-producing *Metschnikowia* yeasts and compare the diverse views on the mechanism(s) underlying their antimicrobial antagonism.

## 2. Taxonomy, Evolution and Taxonomic Identification

### 2.1. The M. pulcherrima Clade

Pulcherrimin-producing *Metschnikowia* strains are common components of the yeast communities that colonise ripening fruits, flowers (nectar), tree sap fluxes and also frequently occur in fruit juices and fermenting wine (e.g., [2,3,6,9,12,18,19,20,21,22,23,24,25,26,27,28,29,30,31,32]). New yeast isolates producing the characteristic maroon-red pulcherrimin halos around their colonies (Figure 1A) are frequently declared to belong to *M. pulcherrima* without taking into account that *M. pulcherrima* is not the only pigment-producing *Metschnikowia* species. Over the past two decades, five additional species (*M. andauensis, M. rubicola, M. shanxiensis, M. sinensis, M. zizyphicola*) were validly described and *M. fructicola*, originally described as a pigment-less species has also turned out to produce pulcherrimin (for a review, see [1]). The intensity of pigment production is variable and highly dependent on the culturing conditions [6,33,34] and probably also on ploidy [35]. The phylogenetic analysis of the barcode sequences of the type strains of the genus clustered these species in a group designated *M. pulcherrima* clade [1,2].

Recently, two additional species, *M*. *persimmonesis* and *M. citriensis* were proposed to accommodate pulcherrimin-producing strains. The taxonomic name *M. persimmonesis* was proposed for a single Korean isolate but without providing a complete taxonomic description [27]. The phylogenetic position of the strain is uncertain because its different rDNA barcode sequences (D1/D2, ITS and 18S) show the highest similarities to sequences of the type strains of different species. *M. citriensis* is based on two strains isolated from citrus leaves [30]. The taxonomic position of these strains is also somewhat obscure, because the authors found them closely related to *M. koreensis* based on the neighbour-joining analysis of the D1/D2 domains of the 26S rRNA genes, but the *M. koreenesis* sequence used in the analysis was a direct GenBank submission amplified from a strain for which no taxonomic description is available. The other closest relatives were strains of three pigment-producing members of the *M. pulcherrima* clade and the non-pigmented *M. chrysoperlae*, but only one sequence used in the phylogenetic analysis represented a type strain. As previous analyses found *M. koreensis* separated by a large phylogenetic distance from the *M. pulcherrima* clade [1,33], the proposed simultaneous close relationship to *M. koreensis* and the *M. pulcherrima* clade needs to be revised or reinforced by the analysis or more sequences. Interestingly, when the ITS sequences are examined, the *M. citriensis* type strain differ more from the other *M. citriensis* strain than from the *M. persimmonesis* type strain. Besides, both the D1/D2 and the ITS sequences were cloned, and a phylogenetic analysis based on cloned sequences can easily be misleading in this group of yeast species because of the very high intragenomic diversity of the rDNA repeats (see below). The formation of spheroidal ascospores is another problematic feature of these isolates. *M. pulcherrima* and its relatives have needle-shape spores [33]. Because of these uncertainties, further examination is required to validate the status of *M. persimmonesis* and *M. citriensis* as distinct species. Nevertheless, most properties of their strains and the results of the sequence analyses indicate taxonomic affinity with the *M. pulcherrima* clade. Many pulcherrimin-producing isolates were not identified at the species level or could not be assigned to any species and were therefore only classified as *Metschnikowia* sp., *M.* aff. *pulcherrima* or *M.* aff. *fructicola*. On the other hand, many strains have been classified into these species without presenting sufficient taxonomic evidence. Since pigmentation is an irrelevant property in most biotechnological processes, the strains isolated for industrial purposes are normally not tested for pulcherrimin production. Therefore and because of the sensitivity of pulcherrimin synthesis to the culturing conditions, it is unknown whether pigmentation is a general ability of all strains of the clade. 

### 2.2. Intragenomic rDNA Repeat Heterogeneity (Lack of rDNA Homogenisation): Limited Applicability of rDNA to Barcoding of Pulcherrimin-Producing Metschnikowia Species

The taxonomic identification of pulcherrimin-producing *Metschnikowia* strains by the examination of morphological and physiological properties is rarely successful because of the shortage of reliable species-specific phenotypic traits [33]. In fact, the delimitation of all non-*pulcherrima* species of the clade was based on barcode sequence differences, mainly on differences between the segments of the multicopy cistrons encoding ribosomal RNAs. These cistrons code for the three major ribosomal RNAs (18S, 5.8S, and 26S rRNA, separated by the internal transcribed sequences ITS1 and ITS2) and are repeated many times in the genomes of all organisms, so that enough rRNA can be produced when demand for ribosomes (protein synthesis) is high. The rDNA repeats are assumed to evolve neutrally [36] and are therefore considered suitable as markers for the phylogenetic differentiation of species and the taxonomic identification of new isolates [37]. The estimated total rDNA repeat number in Fungi varies considerably, ranging from tens to over 1400 copies per genome [38]. In most species, the rDNA repeats form continuous arrays (for a recent review, see [39]) located in one or a few sites per haploid genome. Despite their high copy numbers, the repeats (units) of the arrays have essentially identical sequences. The sequence homogeneity is maintained by a process referred to as concerted evolution or sequence homogenization. In this process, the units of the array evolve “horizontally,” meaning that a non-harmful mutation that arises in one unit can spread to all other units of the array (for reviews, see [40,41,42]). Because of repeat homogenization, the DNA amplified by primers that can hybridize to all repeats is homogeneous in sequence. Thus, the sequencing of the D1/D2 domain of the LSU rRNA gene and the internal transcribed segments ITS1 and ITS2 has become a routine method used for the taxonomic identification of new yeast and fungal isolates (e.g., [43,44,45]). 

This method widely used in yeast taxonomy turned out to have limited applicability in the pulcherrimin-producing *Metschnikowia* yeasts because the amplified D1/D2 domains and ITS segments frequently have ambiguous nucleotides (e.g., [2,5,12,18,20,46,47,48]). It is unknown how widespread the phenomenon is because the ambiguous nucleotides are usually considered sequencing errors to be eliminated from the nascent sequences before the sequence is deposited in a database. An ambiguous nucleotide can be “repaired” by replacing it with the nucleotide corresponding to the highest peak in the chromatogram or by sequencing one DNA molecule (representing one repeat) cloned from the amplified rDNA. However, if the ambiguous nucleotides were indeed sequencing errors, they should be randomly scattered across the sequences. But they occur in particular sites that do not play significant roles in the secondary structure of the transcribed RNA [13]. Both problem-solving strategies result in “clean” sequences but at the cost of concealing the intragenomic diversity and the risk of generating a sequence that does not exist in the genome (when manual correction is applied) or using an accidentally selected member of the diverse repeats (when a cloned fragment is sequenced). Furthermore, both approaches can generate different sequences from the same genome if the analysis is repeated. For example, for the type strain of *M. pulcherrima,* ITS sequences were deposited in databases that have different nucleotides at certain positions (e.g., KY104205, MK394155, JX188179, JX188180). These modes of problem-solving may partially account for the many database sequences (e.g., *Metschnikowia* sp., *Metschnikowia* aff. *pulcherrima*, *Metschnikowia* aff. *fructicola*) that could not be assigned to species because they differed from those of all type strains (e.g., [6,12,17,20,29,46,49]). It is worth mentioning that the delimitation of the species *M. shanxiensis, M. sinensis*, *M. zizyphicola*, *M. persimmonesis* and *M. citriensis* was based on single cloned sequences [19,27,30] without providing information about the homo- or heterogeneity of the rDNA repeats in the genomes of the type strains. 

Why are certain nucleotides ambiguous in the sequences of the amplified rDNA barcode segments? Cloning of individual DNA molecules from amplified D1/D2 and ITS sequences revealed that the type strains of the species *M. andauensis* and *M. fructicola* (both delimited with rDNA sequences containing ambiguous nucleotides [5,18]) have diverse rDNA repeats in their genomes [13,14]. A search for ITS sequences in the genome sequence of the *M. fructicola* type strain (ANFW00000000.2, [15]) further broadened the range of repeat diversity [14]. These sequences differed from one another at the sites where the sequences of the bulk (non-cloned) amplicons had ambiguous nucleotides. So, the presence of these nucleotides was due to the lack of repeat homogenization. Recently, cloned (individual) ITS and D1/D2 sequences were made publicly available in the GenBank database for four additional type strains (*M. pulcherrima*, *M. sinensis*, *M. shanxiensis* and *M. zizyphicola*) of the species group. These sequences also show high intragenomic diversity. Obviously, none of the type strains of these pulcherrimin-producing species has homogenised rDNA repeats. 

### 2.3. The Possible Alternative to rDNA Homogenisation: Birth-and-Death Evolution of Repeats

Why are the repeats not homogenized? Most probably because they are not organized in continuous arrays. A search of the genome sequence (ANFW00000000.2, [15]) of the *M. fructicola* type strain for ITS sequences identified rDNA repeats scattered over the genome [14]. The recently sequenced *Metschnikowia* aff. *pulcherrima* APC 1.2 (ASM421770v1, [17]) strain also has repeats in multiple locations. As homogenization works on continuous arrays [41,42], the scattered rDNA repeats of these strains cannot be (efficiently) homogenized. Recent studies have shown that several repeat families previously thought to have evolved via concerted evolution (homogenization), actually evolve according to a birth-and-death mechanism [40,50]. In this mode of evolution, the balance of two counteracting processes ensures the optimal number of active repeats. New copies of functional units are created by gene duplication (“birth” of repeats) and old copies burdened by accumulating harmful mutations are gradually degraded (“death” of repeats). The new-born repeats provide functional transcripts, whereas the decaying repeats are transcribed into aberrant RNA molecules or become silent. According to a recently proposed model, the rDNA repeats of the *M. pulcherrima* clade strains evolve in a similar way [14]. The exceptionally high diversity of their ITS and D1/D2 sequences is attributable to the simultaneous presence of functional repeats and worn-up repeats inactivated by gradually accumulating mutations. It is assumed that the truncate repeats found in the genome of the *M. fructicola* type strain are in advanced stages of repeat degradation (death). Incomplete rRNA genes can also be found in the genome sequence of the strain APC 1.2 [17]. 

### 2.4. Lack of Clear rDNA Barcode Gaps: Fuzzy Species Boundaries

The ranges of the intragenomic and intergenomic pairwise differences between the cloned *M. andauensis* and *M. fructicola* sequences were practically identical and certain ITS2 clones were found in both type strains [14]. The highest intragenomic pairwise differences (D1/D2: 3.6 and 5.0%; ITS: 14.7%) exceeded considerably the taxonomic thresholds proposed by Vu et al. [51] to discriminate ascomycetous yeast species (D1/D2: 0.59%; ITS: 1.69%) and genera (D1/D2: 2.89%; ITS: 3.69%). The same holds true for the cloned sequences deposited in GenBank for the other four species mentioned above. With the high D1/D2 and ITS intragenomic diversity, the *Metschnikowia* species are exceptional because in other ascomycetes for which relevant data are available either no or much lower levels of intragenomic variation were detected [52,53,54,55,56]. 

**Figure 2:**
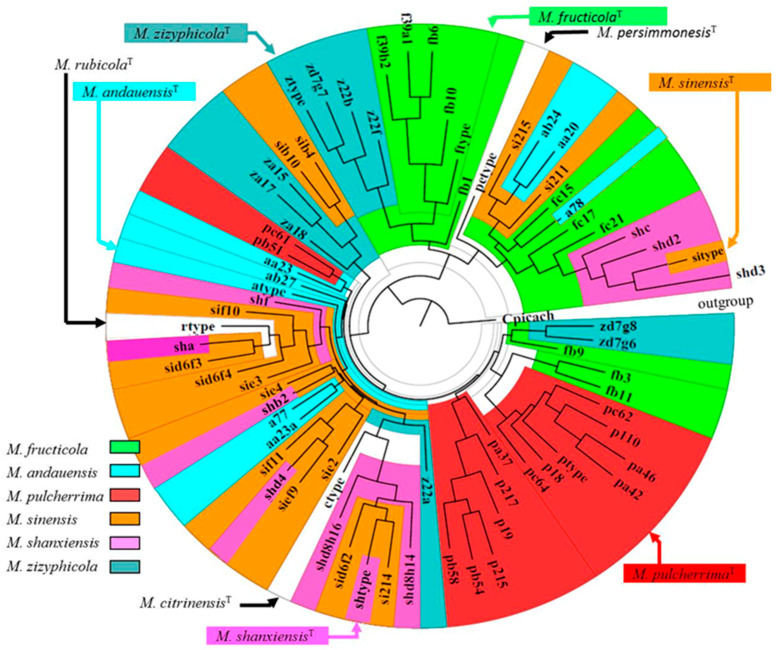
A phylogenetic tree derived from the neighbor-joining analysis of the cloned D1/D2 sequences of the type strains of six pulcherrimin-producing *Metschnikowia* species and the D1/D2 sequences of the type strains available in databases. Outgroup: *Candida (Metschnikowia) picachoensis* (Cpicach). Type-strain sequences are highlighted by arrows. GenBank accession numbers are listed in the supplementary Table S1.

Because of the high intragenomic diversity and the presence of certain sequences in more than one type strain, it is not surprising that no well-separated clusters were formed on the phylogenetic trees and in networks inferred from these sequences [13,14]. Figure 2 shows a tree inferred from the phylogenetic analysis of cloned D1/D2 sequences of the type strains of six species of the *M. pulcherrima* clade, and the D1/D2 sequences of the type strains (among them the *M. andauensis* and *M. fructicola* sequences containing ambiguous nucleotides). None of the species form separate clusters on the tree, indicating that the pulcherrimin-producing *Metschnikowia* type strains are not separated by rDNA barcode gaps. They share a continuous pool of diverse rDNA repeats that are likely to evolve by reticulation [13,14]. For reticulation, the repeats of the strains need to be brought together and then separated. This process requires hybridization. The type strains of *M. pulcherrima*, *M. andauensis* und *M. fructicola* were found to be sexually compatible, capable of hybridization and their hybrids segregated [14]. Thus, the species represented by these type strains are not isolated biologically (reproductively). 

The lack of clear ITS barcoding gaps makes the application of the UNITE database of public fungal ITS sequences to strain identification (https://unite.ut.ee/analysis.php) problematic. UNITE clusters the ITS sequences to create SHs (Species Hypothesis). For each SH, a representative sequence is chosen automatically by computing the consensus sequence of the sequences grouped in it and then finding the best matching sequence of the consensus sequence. In the case of species with high intragenomic rDNA diversity, this procedure can produce disputable results. For example, the sequence (KM213979) selected as representative for the *M. pulcherrima* SH180747.07FU was cloned from the type strain of *M. andauensis*.

The intragenomic diversity of rDNA repeats also poses problems in meta-barcoding analysis (amplicon-based phylotyping) when population diversity is estimated from barcode sequence diversity because the analysis cannot differentiate between sequences representing different strains and sequences representing different repeats of the same strain. In a recent investigation of wine yeast population dynamics, ITS phylotyping was found to over-estimate (overabundance bias) the representation of *Metschnikowia* by one order of magnitude [57]. 

### 2.5. Other Barcode Sequences

Conserved protein-encoding genes (gene segments) are also widely used in molecular taxonomy to differentiate species. These genes usually have single copies in haploid genomes, so the sequences of their amplified fragments are supposed to be free of ambiguous nucleotides if the genome of the organism is haploid. *ACT1* (actin), *RPB2* (RNA polymerase II second largest subunit) *EF1-α* (TEF1; translation elongation factor 1-α) and *EF2 (EFT2;* elongation factor 2) have been sequenced for most type strains of the *M. pulcherrima* clade [2,18,58]. Surprisingly, the *RPB2, EF1*-α and *EF2* database sequences of all type strains contain 1.5–2.5% ambiguous nucleotides [2,57]. Moreover, the “clean” sequences usually do not differ significantly. For example, the type strains of *M. pulcherrima* and *M. fructicola* have identical *ACT1* sequences and their *EF2* sequences differ only by a single substitution. In contrast to this, their *RPB2* sequences are more different and seven out of the ten different positions are ambiguous in one or the other strain. Thus, neither these genes seem to be suitable for reliable barcoding of the species of the clade.

### 2.6. Hybridisation and Chimeric (“Hybrid”) Genomes

The presence of ambiguous (polymorphic) sites in barcode sequences implies that the genes containing these sequences have two or even multiple copies in the genomes. The increase of the copy number can be due to gene (even genome) duplication (followed by divergent evolution of the paralogues) or to hybridization of different strains generating heterozygous diploids or to hybridization followed by postzygotic genome chimerization resulting in segregants of chimeric genomes consisting of mosaics of the genomes of the parental strains. Both genome duplication and postzygotic genome chimerization are well-documented processes in the *Saccharomyces* genus (for recent reviews, see [59,60]). As mentioned above, three type strains of the group are sexually compatible and produce viable hybrids [14]. Genome sequencing also indicates that hybridization can be involved in the evolution of the genomes of these yeasts. The type strain of *M. fructicola* (277) was found to have a significantly larger genome (26 Mb) (ANFW02000000) than certain related species and is highly heterozygous [15,61]. The size of the genomes of AP47 (MTJM01000000) and FL01 (VFXK00000000) is also nearly 26 Mb [15]. In contrast to these genomes, the genomes of APC 1.2 (GCA_004217705) and UCD127 (QBLL01000000) are roughly by one third smaller (15.88 Mb and 17.1 Mb, respectively), and the predicted numbers of their protein-coding genes (5,800 and 5,807) are also much lower than that of 277 (9,674) [15,16,17]. Despite its relatively large genome size, the strain 277 is not diploid because only 5,132 genes out of 8,629 are present in two copies. 228 genes had three or more copies and the rest had only single copies. This strain might have evolved from a hybrid of two strains whose homologous genes differed in sequence, then the hybrid genome underwent a postzygotic genome reduction-chimerization process similar to that characteristic of many *Saccharomyces* chimerical strains (for a review, see [60]) during which large number of genes (>3000) lost their counterparts from one or the other sub-genome. With its chimeric genome, this strain is not an ideal choice to represent a species as its type strain. In contrast to the 277 and AP47 genomes, the genomes of the strains APC 1.2 and UCD127 are by roughly one third smaller (15.88 Mb and 17.1 Mb, respectively), and the predicted numbers of their protein-encoding genes (5,800 and 5,807) are also much lower than that of 277 (8,629) [15,16,17] but even these genomes show considerable levels of heterozygosity [16,17], indicating that they may not be haploid either. 

However, the uncertain phylogenetic relationships of the sequenced strains caused by the obscure species boundaries make it difficult to draw conclusions from genome sequence comparisons. The strain 277 (CBS 8853) is the type strain of the species *M. fructicola* whose delimitation was based on rDNA sequences containing ambiguous nucleotides. The high intragenomic diversity of its D1/D2 and ITS sequences makes it practically indistinguishable from the type strains of the related species (Figure 2). AP47 was originally identified by ITS and D1/D2 sequencing as *Metschnikowia* sp. [22] but genome-sequenced under the name *M. fructicola* [15], although its ITS sequence (FJ919773) was much more similar to sequences cloned from the *M. andauensis* and *M. pulcherrima* type strains (e.g., KM243748; KM209324) and to many *Metschnikowia* sp. and *M.* aff. *pulcherrima* sequences than to the *M. fructicola* type-strain sequences available in databases. The taxonomic affiliation of UCD127 is also uncertain because its D1/D2 rDNA region clustered it with *M. chrysoperlae* whereas its *ACT1* and *TEF1* sequences showed a closer relationship to *M. fructicola* and *M. pulcherrima* [16]. The determination of the taxonomic affiliation of the strain APC 1.2 was attempted by using the UNITE database but because of its multiple diverse ITS sequences it could not be assigned to any species. The authors concluded that “APC 1.2 should properly be designated *M.* aff. *pulcherrima* rather than *M. pulcherrima”,* but for “convenience”, they published the sequence under the name *M. pulcherrima* [17] and use this name for APC 1.2. since then [62]. FL01 was described as the type strain of a novel taxon of incompletely resolved taxonomic status (see above). The fuzzy species boundaries also render it difficult to interpret the experimental results of the investigation of the antimicrobial antagonism of the species and strains of the *M. pulcherrima* clade (see next section).

## 3. Antimicrobial Antagonism

**Figure 3:**
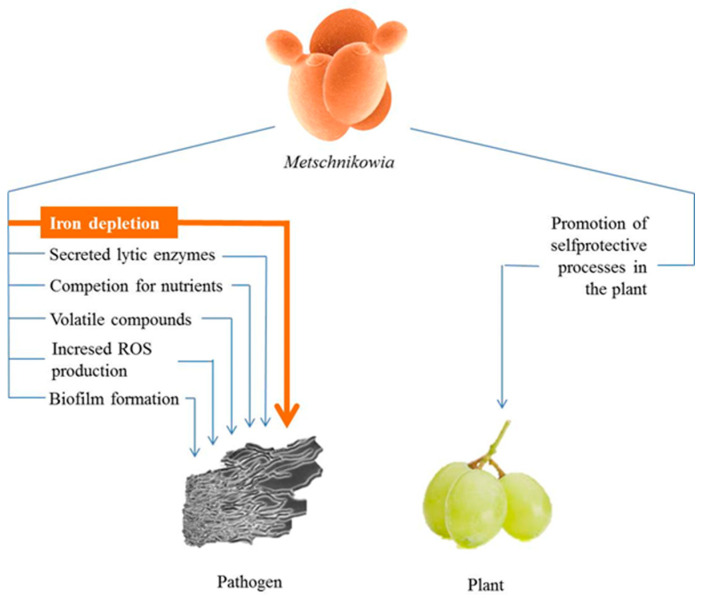
Processes implicated in the antimicrobial antagonism of strains of the *M. pulcherrima* clade.

Due to their antagonistic activity, many *M. pulcherrima* clade strains are considered biological (biocontrol) alternatives to the chemical agents widely used to protect crops and stored agricultural commodities against pathogenic and destructive microorganisms. Antagonism is not an exclusive property of the *M. pulcherrima* clade; strains of many other yeast species can inhibit other microorganisms. Yeasts are known to exert adverse effects on other microorganisms by diverse mechanisms such as competition for nutrients, secretion of cell-wall lytic enzymes and siderophores, release of volatile compounds, production of killer factors, direct physical contact, biofilms formation, etc. and their combinations (for recent reviews, see [62,63,64,65,66,67,68,69,70]). Several of these mechanisms have also been implicated in the antimicrobial activity of the pulcherrimin-producing *Metschnikowia* yeasts (Figure 3, Table 1). This section provides an overview of these mechanisms. 

Because of the lack of clear barcode gaps between the species, and because many strains were not identified at the species level and even if they were, the identification results were often not presented in the publications, the pulcherrimin-producing *Metschnikowia* yeasts will be treated as a continuum (genetically continuous population) in this section. Only strain designations will be used even if the strains were assigned to one or the other species by the authors. In this way, it can be avoided that properties of strains of unknown or insufficiently documented taxonomic positions are misleadingly assigned to species. 

### 3.1. Iron Depletion by Pulcherrimin Production

It had been known since the pioneering work of Beierink that *Metschnikowia* (*Candida, Chlamydozyma, Rhodotorula, Torulopsis*) *pulcherrima* strains produce the maroon-red pigment pulcherrimin (Figure 1A,C) (reviewed in [33]), and later it was also noticed that many strains of the species antagonized other microorganisms (Figure 1B), but the causative relationship between these two properties was experimentally proven only in 2006 [6]. Correlation was noticed between the intensity of the pigment production and the intensity of the antagonism against *Botrytis cinerea* and several other fungi, yeasts and bacteria. Strains producing less pulcherrimin were less effective antagonists and mutants defective in pigment production did not inhibit the growth of *Botrytis* (and other microorganisms) at all. According to one interpretation of the observed correlation, the antagonistic power correlates with the intensity of pulcherrimin production because pulcherrimin is an agent having antimicrobial activity (e.g., [3,9,21,34,69,81]). However, this notion is not based on experimental results and is hard to reconcile with the insolubility of pulcherrimin. The other model relies on the results of the chemical analysis that revealed that pulcherrimin is a chelate of a cyclic dipeptide and ferric ions. According to this model, the sequestration of iron by chelation can be lethal or at least inhibitory to microorganisms that require higher amounts of iron for their cellular processes. Thus, it is not pulcherrimin but the iron deficiency caused by its production through which the *Metschnikowia* strains antagonize other microorganisms [6]. This model was validated by emulation of the inhibitory effect of pulcherrimin production by chelating the ferric ions with tropolone, which also forms a precipitate (ferric tropolone) with ferric ions. Tropolone inhibited the growth of the test microorganisms in the same way as the antagonistic *Metschnikowia* strains [6]. Besides, the supplementation of the medium with excessive amount of ferric ions suppressed the inhibitory effect of both the *Metschnikowia* cells and tropolone. These experiments were carried out with *Metschnikowia* isolates whose exact taxonomic affiliation could not be determined because their D1/D2 sequences differed from those of all type strains of the *M. pulcherrima* clade [6]. Correlation and causative relationship between iron depletion (pulcherrimin production) and antagonism was later observed in many other pulcherrimin-producing *Metschnikowia* strains, including the type strains of the species (e.g., [7,8,10,11,21,71,72,73]).

**Figure 4:**
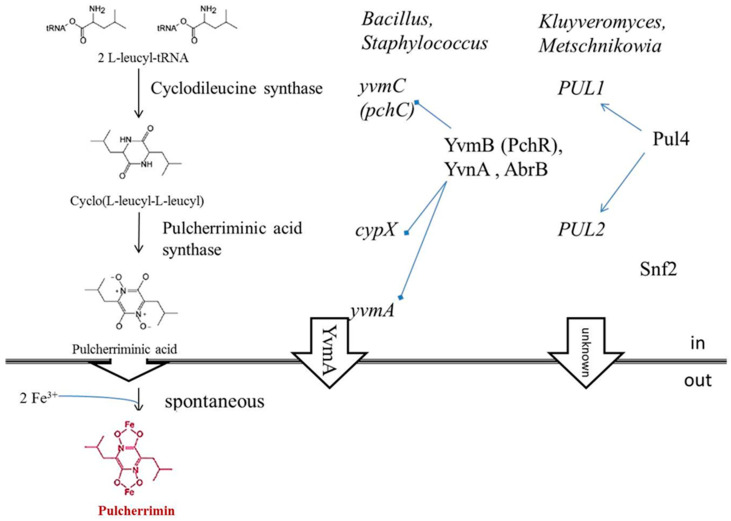
General scheme of pulcherrimin biosynthesis in bacteria and yeasts. Gene names are in italics. For the explanation of symbols and the references, see Section 3.1.

**Figure 5:**
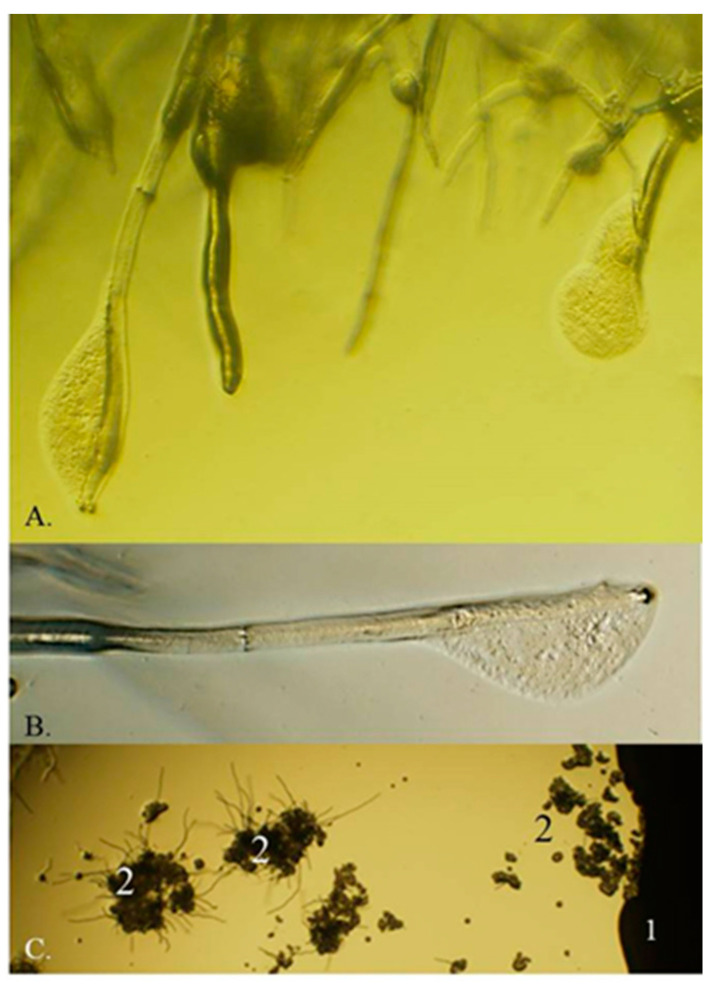
Hyphal lysis and inhibition of germination of *Botrytis* conidia in the inhibition zones surrounding *Metschnikowia* colonies. (**A** and **B**) Lysing and dying hyphal tips. (**C**) Inhibition of the germination of conidia. 1: *Metschnikowia* colony; 2: conidia.

Pulcherrimin is an insoluble iron chelate formed via a non-enzymic reaction between Fe^3+^ and the water-soluble and diffusible pulcherriminic acid secreted by the cells of the antagonistic microorganism. The biosynthesis of pulcherriminic acid has been investigated in detail in *Bacillus subtilis* and *B. licheniformis* and a similar pathway is assumed to operate in yeast cells as well (Figure 4). In *Bacillus*, the Cyclo(L-leucyl-L-leucyl) synthase YvmC/PchC (EC 2.3.2.22) catalyses two molecules of leucyl-tRNA to generate the dipeptide cyclo-L-leucyl-L-leucyl, which is then oxidized by the pulcherriminic acid synthase CypX (EC 1.14.15.13) to pulcherriminic acid. The latter is secreted to the environment where it chelates with ferric ions to form pulcherrimin [82,83]. Homologues of the bacterial genes encoding these enzymes could not be identified in yeast genomes, but two genes (designated *PUL1* and *PUL2*) coding for proteins very different in sequence from their bacterial counterparts could be assigned to both reactions in pulcherrimin-producing *Kluyveromyces* and *Metschnikowia* strains [17,84] (Figure 4). The enzyme-encoding genes are regulated by transcription factors both in bacteria and in yeasts. In *Bacillus*, the YvmB (MarR-like regulator), YvnA and AbrB proteins negatively regulate the process [82,83]. In *K. lactis*, Pul4 acts as a joint regulator of *PUL1* and *PUL2* [84]. Its counterpart was found in the genomes of the *Metschnikowia* strains 277 and APC 1.2. but the function of the *Metschnikowia* Pul4 protein has not yet been investigated [15,17]. Non-pigmented strains can arise by induced [6] and spontaneous [17] mutations as well as by random integration of transforming DNA into the genome [85]. Interestingly, the analysis of a pigment-negative spontaneous mutant of APC 1.2. identified an inactivating mutation in a gene different from the *PUL* genes [17]. Its product turned out to be the counterpart of the non-essential *S. cerevisiae* protein Snf2, the catalytic subunit of the *Saccharomyces* SWI/SNF chromatin remodelling complex [86]. Components of the SWI/SNF complex are not specific transcription factors. This complex contributes to the transcription of many functionally diverse genes by altering the nucleosome structure at the binding sites of the transcription factors (reviewed in [87]). It was proposed that a similar chromatin-remodeling complex also operates in *Metschnikowia*, and the inactivation of its Snf2 subunit prevents the transcription of many *Metschnikowia* genes including *PUL1* and *PUL2* [17]. Within the pigmented zone, the lack of free iron prevents the germination of conidia and inhibits the growth of hyphae of many fungi and the cells of sensitive yeasts and bacteria [6,7] (Figure 5).

Pulcherrimin is sometimes considered a siderophore (e.g., [7,15,63,64,67,84,88,89,90]) although it does not meet the definition. By definition, siderophores (Greek: “iron carrier”) are small, high-affinity iron-chelating compounds secreted by microorganisms to form soluble ferric complexes with the iron available in the environment and then the diffusible siderophore-Fe^3+^ complex moves into the cell using specific membrane receptors and active transport mechanisms [91,92,93]. Pulcherriminic acid meets two of the criteria because it is secreted and binds iron but it does not meet the third criterion because its chelate, the pulcherrimin is a sessile, non-diffusible complex. Thus, neither pulcherriminic acid nor pulcherrimin can serve as an efficient iron carrier. In spite of this, a gene (*PUL3*) was found in *K. lactis* whose activity enabled the cells to acquire iron even after the treatment of the medium with pulcherriminic acid (immobilization of ferric ions in pulcherrimin). Pul3 is an uncharacterized protein but it contains a Major Facilitator Superfamily domain which indicates that it could act as an iron transporter [84]. However, no experimental data prove that Pul3 can transfer the water-insoluble pulcherrimin into the cell. Its substrates could be soluble pulcherrimin derivatives or other types of iron-containing compounds. Remarkably, the transcription of the *PUL3* gene is also under the control of the Pul4 regulator. A gene coding for a Pul3-like protein was also found in the genome sequence of the *Metschnikowia* strain APC 1.2. [17]. Nevertheless, even if pulcherrimin itself is not a siderophore, the *Metschnikowia* cells can produce siderophores. The strain EXF-6812 was found positive in a siderophore test [94].

The anti-microbial antagonism of *Metschnikowia* strains was occasionally proposed to be due killer toxins (e.g., [25,95,96,97,98]). However, no clear distinction was made between killer-induced inhibition and non-killer antagonism in these studies, and it can be inferred from the context that the term killer activity was used synonymously with the term antagonistic activity (e.g., antagonism associated with pulcherrimin production) which can be misleading. All killer toxins studied so far have been found to be proteins or glycoproteins with molecular weights ranging from 5000–10,000 to 100,000 Da or even greater in a few cases (for a review, see [99]).

Although iron depletion is a robust inhibitory mechanism, data are available that indicate that other mechanisms can also contribute to the antagonistic activity of the *Metschnikowia* cells. Manso et al. [75] found that the antagonistic activity of the strain NCYC 3728 (PBC-2) was independent of the intensity of pulcherrimin production. The pigment-less mutant snf2 retained some antagonistic activity despite producing no pulcherrimin [17]. In contrast to this observation, other pigmentless mutants lost the antagonistic activity completely [6,85]. The difference can be attributed to the use of different definitions of antagonism, different testing methods or different defects in different genes but also to mechanisms different from iron depletion (Figure 3). 

### 3.2. Competition for Nutrients

Competition for nutrients is perhaps the most widespread mechanism by which a microorganism can adversely affect the growth of another microorganism. The organism that utilizes a nutrient faster or more efficiently than its rival (competitor) suppresses the propagation of the latter. Certain *Metschnikowia* strains were observed to adversely affect the growth of other microorganism by consuming certain nutrients which are required by both of them. Piano et al. [74] found that addition of nitrates reduced or even totally suppressed the biocontrol activity of the antagonistic strains 2.33 and 4.4 against *B. cinerea* on apple. Similar suppression of the antagonistic effect was achieved with glucose and fructose (but not sucrose) on mango fruits [31]. Addition of these two sugars into the wounds of fruits infected with an antagonistic soil-born *Metchnikowia* strain and the post-harvest pathogen *Colletotrichum gloeosporioides* drastically increased the size of the disease lesions around the wounds. From the observed reduction of the antagonistic efficiency by nutrient supplementation it was inferred that these *Metschnikowia* strains suppressed the growth of the pathogen by fast depletion the fruit surface/wounds of certain nitrogen and carbon sources.

### 3.3. Secretion of Enzymes

Microorganisms can exert harmful effects by secretion of enzymes that damage the walls or other components of the cells of other microorganisms. Several strains of the *M. pulcherrima* clade were found able to secrete extracellular lytic enzymes, such as chitinase and glucanase (e.g., [10,11,75,76,77,100,101]) but the significance of this secretion is disputable in certain cases. For example, the antagonistic strain MACH1 secreted chitinase in various media including apple juice [76]. In contrast, the strain FL01 did not respond to the presence of the pathogen *Geotrichum citri-aurantii* with increased β-1, 3-glucanase (GLU) and chitinase (CHI) production [73]. AP47 showed increased chitinase activity when grown in a minimal medium with *Monilinia fructicola* cell wall preparation as the sole carbon source [101]. The strain NCYC 3728 (PBC-2) secreted chitinase after 5 and 7 days of incubation in YNB (yeast nitrogen base) medium supplemented with *P. expansum* cell wall [75]. In a recent study, seven strains showed modest extracellular chitinase activities [72]. In contrast, Parafati et al. [10] detected neither β-1,3-glucanase nor chitinase secretion in a different collection of *Metschnikowia* strains. Even closely related strains can differ in the production of extracellularly measurable chitinase activity: FL01 showed chitinase activity but no extracellular enzyme activity was detected in the culture of FL02 [11]. In the cells of the strain 277 the chitinase gene *CHI* was found upregulated not only by the presence of *P. digitatum* hyphae but also by the presence of grapefruit peel tissue [61]. The same study identified 153 genes of highly diverse functions whose expression changed in the presence of the hyphae but their involvement in the antagonistic activity of the yeast cells was not verified experimentally. Thus, the wall lytic enzymes can contribute to the overall antifungal effect of certain strains, but they are unlikely to play major roles. Moreover, the production of these enzymes may not be specific for antifungal antagonism. As pointed out by Pretcher et al. [72], “the involvement of lytic enzymes in antagonistic reactions remains essentially unclear and may depend on experimental and environmental conditions.” The strain PO1C004 was found to release DNAse on PDA agar supplemented with DNA [26].

### 3.4. Release of Volatile Compounds

The antagonistic effect of volatile compounds released by *Metschnikowia* cells was demonstrated both by in-vitro assays and on grapes infected with *Botrytis* [10,77]. Parafati et al. [10] detected drastic reduction of rotting on *Botrytis*-infected grape berries in the presence of agar cultures of the *Metschnikowia* strain MPR3. Oro et al. [77] observed correlation between the production of ethyl acetate by the strain Disva 267 and the negative effect of the strain on the growth rate of certain filamentous fungi under laboratory conditions. When cultivated in a liquid medium, Disva 267 produced ethyl acetate, a volatile organic compound commonly found in food and beverages and known for its antimicrobial effect. In a different experiment, the strain was grown on agar plates covered with plates turned upside down. The fungi inoculated on the latter plates grew more slowly than on the control plates. The authors concluded that the reduction of growth of the mycelium might have been attributed to ethyl acetate released by the yeast cells. In contrast, the strain FL01 did not produce volatile organic compounds with inhibitory effect on *G. citri-aurantii* [73]. Interestingly, the same strain reduced the growth rate of *P. digitatum* and *P. italicum* by volatile compounds in laboratory tests [64]. Thus, the inhibition of fungi by yeast-borne volatile compounds appears to be strain and target dependent. Nevertheless, the inhibition zones seen on agar media around the *Metschnikowia* colonies are hardly attributable to volatile compounds.

### 3.5. Biofilm Formation and Adhesion to the Plant Surface and Fungal Hyphae

Biofilms formed by aggregated communities of yeast cells on injuries of the plant surface can provide a physical barrier that deters the expansion of the hyphae of the pathogenic fungi. Correlation between biofilm formation and colonization capacity on table grape wounds was observed for seven *Metschnikowia* strains tested by Parafati el al. [10]. In a different study, the increased pulcherrimin production of the strain FL01was accompanied with increased biofilm formation [11]. This observation led to the hypothesis that the secreted pulcherriminic acid protects the plant tissues against the pathogens not only by sequestering the iron but also by promoting the yeast cells to form films which hold up the fungal invasion [11,73]. For the applicability of an antagonistic strain to bioprotection, it is also important that its cells adhere to the surface of the plant to be protected. The cells of strain FL01 were found to tightly adhere to the surface of the wounds of citrus fruits [73]. In a group of ten isolates, Pawlikowska et al. [12] observed considerable diversity in the efficiency of cells to adhere to glass (hydrophilic) and polypropylene (hydrophobic) surfaces, but the efficiency of adherence did not correlate with the antagonistic power. The strains FL01 and FL02 were found to adhere also to the hyphae of *P. digitatum* [64], suggesting that the direct physical contact with the target organism may also play a role in the antagonism of certain strains.

### 3.6. Indirect Antagonism through Modulation of Plant Defence Response

Numerous studies demonstrated that plants respond to exposure to various biotic and abiotic stresses by elevated production of reactive oxygen species (ROS) (reviewed in [102]) and this response can play an important role in the defense mechanism of the plant against the pathogens (e.g., [103]). When tested on wounded fruits, the *Metschnikowia* strain 277 produced high levels of ROS and (like certain other microorganisms) also triggered elevated ROS production in the wounded plant tissue [78]. It was suggested that these two processes may jointly contribute to the biocontrol activity of this antagonistic strain [79]. In line with this assumption, the transcription analysis of grapefruit peel treated with the same strain detected a decrease in the expression levels of the plant genes encoding ROS-detoxifying enzymes [80]. When implicating the elevation of ROS in defending the plant against the attacking microorganism, it should be taken into account that both sides of the barricade can apply elevated ROS production. Not just the plant employs ROS production in defense against the fungal attack but even the fungus can produce ROS during infection to damage the plant tissue [103]. For example, the conidial cells of *B. cinerea* produce hydrogen peroxide to destruct the host plant cells [104] because the accumulation of ROS also results in serious damage to biological macromolecules within the plant cell, leading to metabolic dysfunction and eventually cell death (reviewed in [105]).

## 4. Concluding Remarks and Perspectives

Because of their non-homogenized rDNA repeats and the frequent heterozygosity (nucleotide polymorphism) of their protein-encoding barcode genes, the type strains of the species of the *M. pulcherrima* clade are not separated by clear barcode gaps. Certain type strains were found sexually compatible (producing viable “interspecies” hybrids) and the strains whose genomes have been sequenced are highly heterozygous and chimeric, indicating that they may have evolved from alloploid hybrids. The fuzzy species boundaries make the taxonomic identification of the pulcherrimin-producing strains difficult or even impossible. 

The uncertainty of species boundaries further raises doubts as to how correct the definition of the species within the clade is from a phylogenetic point of view. As pointed out by Lachance [1], “the clade is in a bad need of an expert taxonomic study”. 

Although iron sequestration by pulcherrimin production seems to be a robust mechanism in the antimicrobial antagonism of these yeasts, there is a considerable debate about the involvement of other mechanisms. As different laboratories used different experimental approaches to study the mechanisms, the relative contribution of the different mechanisms and their interplay is largely unknown and need to be studied and elucidated in much more detail. Although the antagonism studies were usually performed with strains assigned by the authors to one or the other of the species, the disputable taxonomic division of the clade makes it is difficult to ascertain which, if any, is species-specific. 

## Figures and Tables

**Table 1:** Mechanisms implicated in the antimicrobial antagonisms of *M. pulcherrima* clade strains.

Mechanism ^1,2^	Strains	References
Iron immobilisation by pulcherrimin production	02.4.3.38, 02.11.1.21, 7.3.37, 17.1.IV, 17.3.1, 152, 160, 192, 2305, 446, 523, 648, APC 1.2, CBS 610NT, FL01, MACH1, MPR3, Msp50, Msp51, UMY12, UMY14, UMY15	[6,7,8,9,10,11,17,21,71,72,73]
Competition for nitrogen source	2.33, 4.4	[74]
Competition for carbon source	Anonymous	[31]
Chitinase secretion	AP47, FL01, MACH1, NCYC 3728 (PBC-2)	[11,75,76]
Chitinase gene up-regulation	277 (CBS 8853)	[61]
DNAse secretion	PO1C004	[26]
Release of volatile compound(s)	FL01, MPR3	[10,64]
Release of ethyl acetate	Disva 267	[77]
Biofilm formation	FL01, MPR3, six anonymous strains	[10,11]
Adherence to plant surface	FL01	[73]
Adherence to fungal hyphae	FL01, FL02	[64]
Increased ROS production	277 (CBS 8853)	[78]
Induction of increased ROS production in plant tissue	277 (CBS 8853)	[78,79,80]
Unknown, independent of pulcherrimin production	NCYC 3728 (PBC-2), snf2	[17,75]
Unknown	KIOM_G15050	[27]

^1^ Experimentally verified or hypothesized on the basis of correlation with antagonistic effect. ^2^ May not be the main or the sole mechanism.

## Supplementary Materials

The following are available online at https://www.mdpi.com/2076-2607/8/7/1029/s1. Table S1: Accession numbers of D1/D2 sequences.
