# Supplementary material for: Metschnikowia pulcherrima and Related Pulcherrimin-Producing Yeasts: Fuzzy Species Boundaries and Complex Antimicrobial Antagonism
Source: Microorganisms. 2020 Jul 12;8(7):1029. doi: 10.3390/microorganisms8071029 (PMC7409158; doi:10.3390/microorganisms8071029)
Supplement: Supplementary file 1 [file microorganisms-08-01029-s001.pdf]

Table S1. Accession numbers of D1/D2 sequences

Designation	Mode of sequencing[1]	Accession number
M. andauensis		
atype (CBS 10809^T)	direct	AJ745110
a77	cloned	KC411953
a78	cloned	KC411954
aa20	cloned	KC411955
aa23	cloned	KC411956
aa23a	cloned	KC411957
ab24	cloned	KC411958
ab27	cloned	KC411959
M. fructicola		
ftype (CBS 8853^T)	direct	AF360542
fb1	cloned	KC411962
fb3	cloned	KC411963
fb6	cloned	KC411964
fb9	cloned	KC411965
fb10	cloned	KC411966
fb11	cloned	KC411967
fc15	cloned	KC411968
fc17	cloned	KC411969
fc21	cloned	KC411970
f39a1	cloned	KC411960
f39b2	cloned	KC411961
M. pulcherrima		
ptype (CBS 5833^T)	direct	U45736
p18 (1mut8)	cloned	KM249361
p19 (1mut9)	cloned	KM249362
p110 (1mut10)	cloned	KM249363
p215 (2mut15)	cloned	KM249364
p217 (2mut17)	cloned	KM249365
pa37	cloned	KM249366
pa42	cloned	KM249367
pa46	cloned	KM249368
pb51	cloned	KM249369
pb54	cloned	KM249370
pb58	cloned	KM249371
pc61	cloned	KM249372
pc62	cloned	KM249373
pc64	cloned	KM249374
M. shanxiensis		
shtype (CBS 10359^T)	cloned	DQ367883
shd8h14	cloned	KM350705
shd8h16	cloned	KM350706
shb2	cloned	KM350707

shd2	cloned	KM350708
Shd3d3	cloned	KM350709
Shd4	cloned	KM350710
sha	cloned	KM350711
shc	cloned	KM350712
shf	cloned	KM350713
M. sinensis		
sitype (CBS 10357^T)	cloned	DQ367881
sid6f2	cloned	KM275352
sid6f3	cloned	KM275353
sid6f4	cloned	KM275354
sib4	cloned	KM275355
sib10	cloned	KM275356
sie2	cloned	KM275357
sie3	cloned	KM275358
sie4	cloned	KM275359
sif9	cloned	KM275360
sif10	cloned	KM275361
sif11	cloned	KM275362
si21-1 (21-1)	cloned	KM275363
si214 (21-4)	cloned	KM275364
si215 (21-5)	cloned	KM275365
M. zizyphicola		
ztype (CBS 10358^T)	cloned	DQ367882
z d7g6	cloned	KM275366
zd7g7	cloned	KM275367
z d7g8	cloned	KM275368
za15	cloned	KM275369
za17	cloned	KM275370
za18	cloned	KM275371
z22a	cloned	KM275372
z22b	cloned	KM275373
z22f	cloned	KM275374
M. citriensis		
ctype (FL01^T)	cloned	MF538699
M. persimmonesis		
petype (KCTC 12991BP^T)	cloned	MF446618
M. rubicola		
rtype (NRRL Y-6064^T)	cloned	MG050901
Candida (Metschnikowia) picachoensis		

Cpicach (CBS 9804^T)	direct	AY452039

[1] direct: DNA amplified from genomic DNA; cloned: DNA cloned from amplified DNA
